# Modeling and Measurement of Sustained Loading and Temperature-Dependent Deformation of Carbon Fiber-Reinforced Polymer Bonded to Concrete

**DOI:** 10.3390/ma8020435

**Published:** 2015-01-29

**Authors:** Yoseok Jeong, Jaeha Lee, WooSeok Kim

**Affiliations:** 1 Department of Civil Engineering, Chungnam National University, Daejeon 305-764, Korea; E-Mail: yosoksi@gmail.com; 2 Department of Civil Engineering, Korea Maritime and Ocean University, Busan 606-794, Korea; E-Mail: jaeha@kmou.ac.kr

**Keywords:** debonding, externally bonded, fiber-reinforced polymer, stress redistribution, creep, temperature

## Abstract

This paper aims at presenting the effects of short-term sustained load and temperature on time-dependent deformation of carbon fiber-reinforced polymer (CFRP) bonded to concrete and pull-off strength at room temperature after the sustained loading period. The approach involves experimental and numerical analysis. Single-lap shear specimens were used to evaluate temperature and short-term sustained loading effects on time-dependent behavior under sustained loading and debonding behavior under pull-off loading after a sustained loading period. The numerical model was parameterized with experiments on the concrete, FRP, and epoxy. Good correlation was seen between the numerical results and single-lap shear experiments. Sensitivity studies shed light on the influence of temperature, epoxy modulus, and epoxy thickness on the redistribution of interfacial shear stress during sustained loading. This investigation confirms the hypothesis that interfacial stress redistribution can occur due to sustained load and elevated temperature and its effect can be significant.

## 1. Introduction

The need for rehabilitating and strengthening deteriorated reinforced concrete (RC) structures is increasing with the continued aging of civil infrastructure. Fiber reinforced polymer (FRP) bonded repairs are widely used as strengthening and retrofitting systems for civil infrastructure. An increasing amount of research has addressed bond strength after various aggressive environmental exposures [1,2,3]. Nevertheless, it is necessary to verify the durability of retrofit systems in the face of time and temperature. Jia *et al.* [4] studied the durability of externally bonded glass FRP (GFRP) reinforced concrete beams subjected to a constant four-point flexural load under different conditions. A severe decrease in interfacial fracture energy is noted for specimens subjected to cycles of dry freeze/thaw (−18 to 25 °C) and elevated temperature/dry (50–60 °C) conditions. Beside the reduction in fracture energy, a shift in bond behavior mode has been observed before and after environmental conditioning exposure such as elevated temperature or moisture [5,6,7]. The elevated temperatures have a significant effect on the bond behavior of FRP-to-concrete interfaces in terms of thermal stress. When temperature exceeds the glass transition temperature, the ultimate load decreases, which is attributable to degradation in the interfacial bond. However, the ultimate load of the bonded joint was found to increase before the temperature reached the glass transition temperature [8,9]. Meshgin *et al.* [10] researched the time-dependent behavior of epoxy at the interface between the concrete and carbon FRP (CFRP) sheet under 23 °C ambient temperature. The experimental results showed that long-term shear behavior of the epoxy is dependent on the epoxy curing time-before-loading. Mazzotti and Savoia [11] investigated the residual strength of double-lap shear specimens with externally bonded CFRP plates after a 5-year period of sustained loading at 50% of initial pull-off strength and 20 °C ambient temperature. Significant strain redistribution along the CFRP plate was observed over time. An increase in pull-off strength, beyond that expected due to concrete aging, occurred as well. The research is closing the gap which started with the initial work of Plevris and Triantafillou [12]. To the knowledge of the authors, it is still necessary to investigate the time-dependent behavior and durability issues of externally bonded FRP systems in depth by means of an experimental and numerical approach.

The overall objective of the present investigation is to determine the combined effects of sustained loading and temperature on the lap-shear bond behavior of CFRP sheets bonded to concrete. In this paper, a numerical model of the bond problem is developed, validated with preliminary experimental results, and used to explore the effects of various bonded-joint parameters on the distribution and redistribution of stresses in the FRP, epoxy, and concrete substrate.

## 2. Experimental Investigation

### 2.1. Single-Lap Shear Test

**Figure 1:**
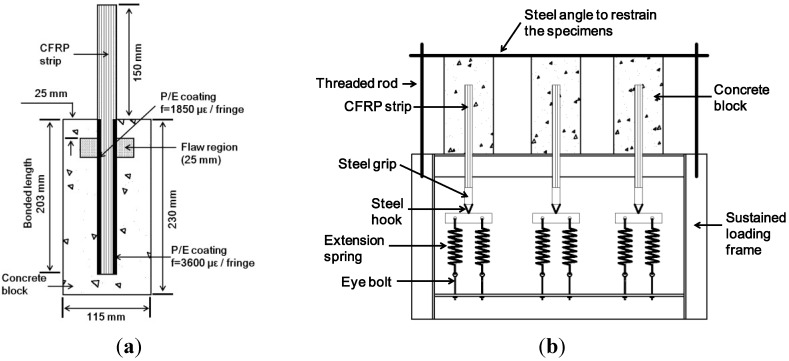
(**a**) Typical single-lap specimens; (**b**) sustained loading set-up.

The experiments used for model validation have been reported in detail previously [13], but are briefly summarized here for completeness. The experiments essentially provided data on the time-dependent distribution of strain in single-lap shear specimens under sustained loads and two different temperatures, although only the room-temperature results are considered presently for model validation. Residual pull-off strength was measured after the completion of the sustained loading period. Single-lap shear specimens were fabricated using concrete blocks (115 mm × 115 mm × 230 mm). A 25 mm wide single ply of unidirectional CFRP sheet was saturated with epoxy and bonded to concrete block using a wet layup procedure. The mechanical properties of the materials used in the model are given in Table 1. Material properties used in the numerical program were obtained from experiments except for the CFRP sheet, whose properties were provided by the manufacturer as shown in Table 1. Figure 1a describes the specimens, some of which were made with intentional bond flaws consisting of a thin polymer releasing film. A narrow strip of birefringent polymer called photoelastic (P/E) coating was bonded to the surface of the CFRP sheet to allow the monitoring of longitudinal strain distribution with a polariscope during sustained loading. A total of 16 single-lap shear specimens were fabricated, allowing for 2 repetitions at each test condition as shown in Table 2. The creep specimen identification scheme in Table 2 consists of a three-part code, A-B-C-n, where A represents the existence of a flaw (NF for no flaw, F for flaw), B represents creep test temperature (RT for room temperature (23 °C), HT for high temperature (40 °C)) and C represents loading conditions (C for control specimen (no sustained load), SL for sustained load).

**Table 1:** Mechanical properties of concrete, epoxy and CFRP for numerical simulations.

Material Properties	Plain Concrete	Epoxy		CFRP *
		23 °C	40 °C	
Modulus of elasticity, GPa	22	3.46	2.99	95.8
Poisson’s Ratio	0.2	0.36	0.36	0.2
Tensile strength, MPa	3.2	71.2	–	986
Compressive strength, MPa	32	–	–	–
Ultimate strain, %	–	–	–	1
Thickness, mm	–	–	–	1

* Provided by the manufacturer [14].

**Table 2:** Specimens used during experimental program.

Specimen ID	Samples	Flaw (Yes/No)	Temperature at Sustained Loading (°C)	Sustained Loading Period (h)
NF-HT-SL	2	No	40	87
NF-HT-C	2			–
NF-RT-SL	2	No	23	87
NF-RT-C	2			–
F-HT-SL	2	Yes	40	87
F-HT-C	2			–
F-RT-SL	2	Yes	23	87
F-RT-C	2			–

**Figure 2:**
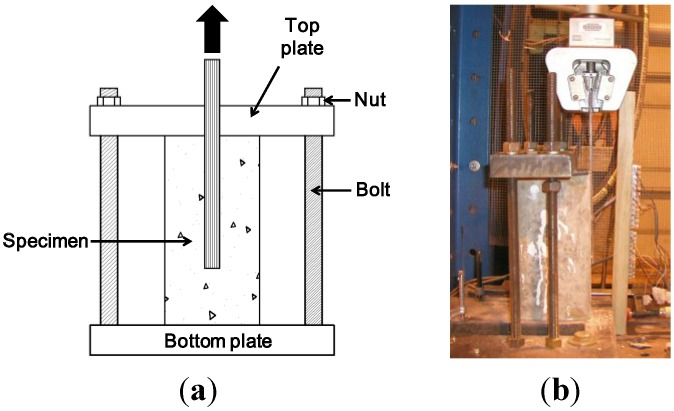
Pull-off test set-up. (**a**) schematic; (**b**) photograph.

For specimens with sustained loading, the load level was selected to be 2.96 kN, which corresponds to a longitudinal strain of 0.12% in the overhung portion of the CFRP sheet. The sustained loading set-up consists of a steel frame with springs for maintaining a constant force on the CFRP as shown in Figure 1b. Once the sustained load was applied, the specimens were stored at room temperature, and at high temperature were monitored for 87 h (5220 min or 3.6 days). Following sustained loading, the specimens were unloaded and then subjected to pull-off tests at room temperature as shown in Figure 2. Two single-lap specimens of each type and test condition were tested.

### 2.2. Epoxy Creep Test

Tensile properties of neat epoxy were measured using at room-temperature (RT, 23 °C) and at high temperature (HT, 40 °C). These results are listed in Table 1. Tensile creep tests of the epoxy were conducted using a lever-arm creep test frame for room-temperature (RT) tests at 23 °C and a dynamic mechanical analysis (DMA) tester for the high temperature (HT) tests at 40 °C. For the RT creep test, the epoxy was cured for seven days at room temperature prior to testing (similar to the thermal history of the single-lap specimens when the sustained loading commenced) and the applied stress was 10 MPa. For the HT creep test, the epoxy was cured for seven days at 40 °C prior to running the creep test and the applied stress was 1 MPa. Strain was measured for approximately 5500 min (3.8 days) during the creep tests.

**Figure 3:**
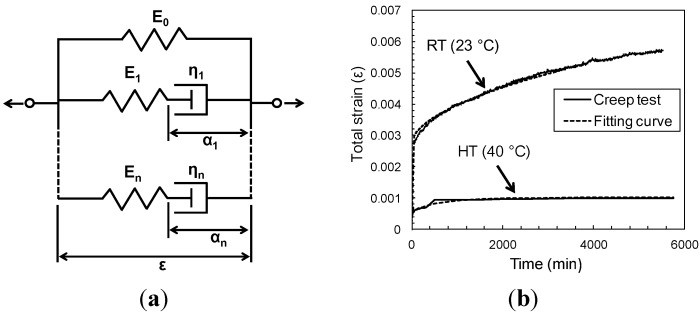
(**a**) Generalized Maxwell model; (**b**) Fitted-curve to the RT/10 MPa and HT/1 MPa creep test data of epoxy.

Based on the creep test results, the viscoelastic properties are determined for numerical study. Over the years a number of theories have been proposed that epoxy polymers are viscoelastic in nature and exhibit creep under constant stress [15,16,17]. The rheological model can describe the viscoelastic materials using springs and dashpots in series or parallel [18]. Relaxation modulus or relaxation test data of viscoelastic materials are required for the determination of the rheological Maxwell models as shown in Figure 3a. The notations *E* and η represent the elastic modulus and the coefficient of viscosity for the Maxwell model. However, because of the limited experimental data available to calibrate relaxation modulus, it is difficult to run a constant-strain relaxation test on stiff materials [19]. In this case, the relaxation modulus can be obtained from the creep compliance, which corresponds to the strain caused by a unit sustained stress through an interconversion. In general, this is achieved through the following expression [20]:

(1) $$\int_{0}^{t}J(u)R(t-u)\text{d}u=t\;\text{for}\;t\;>\;0$$

where *R*(*u*) is relaxation modulus; and *J*(*u*) is creep compliance.

Equation (1) presents a Volterra integral that is solved numerically or analytically using the Laplace transform. Methods of interconversion between relaxation modulus and creep compliance for viscoelastic materials are proposed and well-illustrated in the literature [21]. When the relaxation modulus obtained by numerically solving Equation (1) based on creep compliance is assumed to be a generalized Maxwell solid, the relaxation modulus is typically modeled with a Prony series of the dimensionless relaxation modulus as follows [22]:

(2) $$R(t)=1-\sum_{i=1}^{n}P_{i}\left(1-e^{-t/\tau_{i}}\right)$$

where *P_i_*, τ*_i_* and *n* are material constants defined by the curve fit. Table 3 lists Prony parameters used to predict the time-dependent behavior of a single-lap shear specimen. The time variation of the normalized relaxation modulus of materials used in the numerical program is shown in Figure 3b along with its curve-fit approximation. The Prony series approximations of the relaxation modulus, which appear in Figure 3b, show very good agreement with the given modulus.

**Table 3:** Coefficients of Prony series used in creep modeling.

Specimen ID	*P*_1_	τ_1_ (min)	*P*_2_	τ_2_ (min)	*P*_3_	τ_3_ (min)
Epoxy-HT	0.43	6.45	0.25	353	0.00065	3500
Epoxy-RT	0.11	95	0.16	646	0.41	4196

## 3. Numerical Investigation

### 3.1. Finite Element Model of Epoxy Creep Test

Time-dependent finite element analyses using ABAQUS 6.11-1 [22] were also performed to ensure proper modeling of the creep behavior of epoxy. The experimental creep test specimen was modeled using a 2-D plane stress analysis since the specimen is predominantly in a uniaxial state of stress in the region of strain measurement. In this simulation, the epoxy coupon is represented by the continuum plane stress element (CPS4R), a plane element with four nodes that allows the creep to be modeled as shown in Equation (1).

### 3.2. Finite Element Model of Single-Lap Shear Test

**Figure 4:**
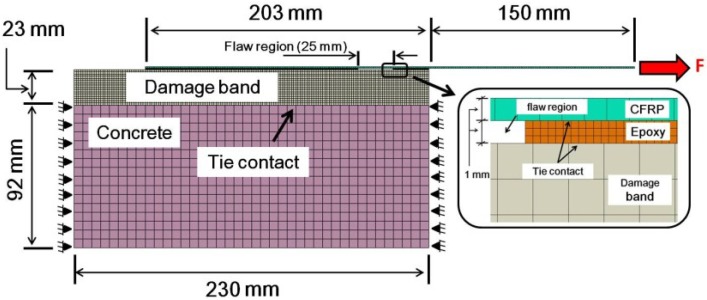
Finite element mesh used for numerical simulations.

Time-dependent nonlinear finite element analyses were performed using ABAQUS 6.11-1 to simulate the sustained loading and pull-off experiments. The finite element mesh used to model the single-lap specimen is shown in Figure 4. The test setup was modeled using a 2-D plane strain analysis. Note that the plane strain conditions dominate when the material surrounding the crack tip provides constraint, as in the present case [23]. Concrete, epoxy, and CFRP were modeled using a continuum plane strain element (CPE4R). Refined meshes were used for accurately simulating the crack band, epoxy and CFRP. Between meshes with different densities, rigidly tied contacts were used. With this technique, each of the nodes on the refined mesh has the same displacement as the point on the adjoining coarse mesh. Normal and shear stresses can therefore be modeled along the entire tie interaction. The material properties used to simulate the specimens are shown in Table 1. The dimension of the damage band was selected following recommendations by Bažant and Planas [24]. During cracking of the concrete, damage localization occurs in a crack-band width *h_c_* = 3*d_a_*, where *d_a_* = maximum aggregate size. It is also important to mention that numerical simulations reported by Coronado and Lopez [25] indicate that variations of the crack-band thickness from 3*d_a_* to 0.25*d_a_* have a minor effect on the overall numerical results.

### 3.3. Material Constitutive Behavior

#### 3.3.1. Model for the Concrete and Damage Band

**Figure 5:**
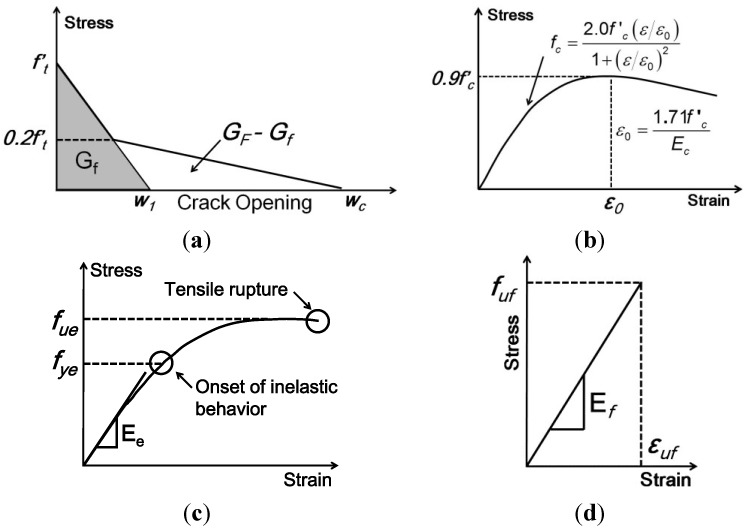
Material constitutive behavior. (**a**) Concrete crack band constitutive behavior; (**b**) Concrete compressive stress-strain behavior; (**c**) Epoxy tensile stress-strain behavior; (**d**) FRP tensile stress-strain behavior.

A plastic damage model was used in order to predict the constitutive behavior of concrete in the damage band. In this approach, it is assumed that tensile cracking and compressive crushing are the main failure modes. Both phenomena are the result of microcracking which can be interpreted as a local damage effect controlled by a yield function, which defines their onset and evolution [24]. Details of formulation are given by Lubliner *et al.* [26] and Lee and Fenves [27]. To specify the post-peak tension failure behavior of concrete, the fracture energy method was used. The softening curve of the crack band under uniaxial tension is shown Figure 5a, where *f'_t_* is the stress determining the onset microcracking, *G_F_* is total external energy supply, per unit area, required to break a Mode I (crack opening) in concrete, *G_f_* is defined as the size-effect fracture energy, and *w_c_* is the crack opening.

The stress-strain curve of concrete under uniaxial compression follows the mathematical model proposed by Todeschini *et al.* [28] and shown in Figure 5b, where *E_c_* is the modulus of elasticity and *f'_c_* is the compressive strength. Values used for these two properties are listed in Table 1. The fracture mechanics properties used in the FE model were obtained from Coronado and Lopez [25] and are shown in Table 4.

**Table 4:** Fracture mechanics properties for concrete crack band.

*f'_t_* (MPa)	*w_1_* (μm)	*G_f_* (N/m)	*G_F_* (N/m)	*w_c_* (mm)
2.62	58.04	76.13	190.32	0.073

#### 3.3.2. Epoxy Model

Two types of constitutive behavior of epoxy are incorporated in this numerical simulation: an elastic-plastic model with strain hardening for quasi-static response during pull-off testing in Figure 5c, and a generalized Maxwell model defined for epoxy creep during sustained loading in Equation (1). In order to define such a behavior, the entire tensile stress-strain curve is provided as input to the finite element analysis, including the modulus of elasticity, *E_e_*, the Poisson’s ratio, ν*_e_*, and the yield strength, *f_ye_*. The stress-strain curve used for the elastic-plastic model was obtained experimentally by Coronado [29] and reported as specimen E-1A. Constitutive laws describing the full range of multiaxial creep behavior for polymers can be very complicated. However, the generalized Maxwell creep model is attractive for its simplicity in cases where the stress state remains essentially constant and low in relation to the yield strength of the polymer.

#### 3.3.3. CFRP Model

In longitudinal tension, the CFRP strip is assumed to behave linear-elastically up to the failure stress (*f_uf_*) and strain (ε*_uf_*) as shown in Figure 5d. At the failure point, the material itself loses all its tensile strength. The other parameters used to model CFRP behavior are the modulus of elasticity (*E_f_*), and Poisson’s ratio (ν*_f_*).

### 3.4. Comparison of Predictions and Experimental Results

#### 3.4.1. Epoxy Creep Test

**Figure 6:**
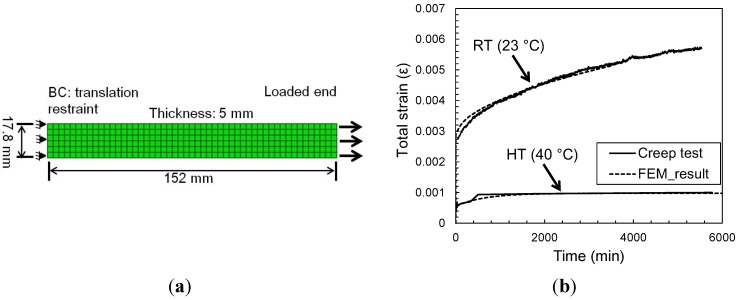
Creep response of epoxy coupons. (**a**) Finite element model of epoxy creep test; (**b**) comparison between experimental and numerical strain variation during creep tests under 10 MPa for room temperature (RT) and 1 MPa for high temperature (HT) conditions.

Figure 6 shows the total strain *versus* time results from the finite element model and the experiment. Good agreement is observed for the entire time. The validated epoxy creep model is subsequently used to numerically evaluate the creep behavior of a single-lap specimen with varying geometric and material parameters for the FRP/concrete joint.

#### 3.4.2. Single-Lap Shear Test

##### Sustained Loading Period

**Figure 7:**
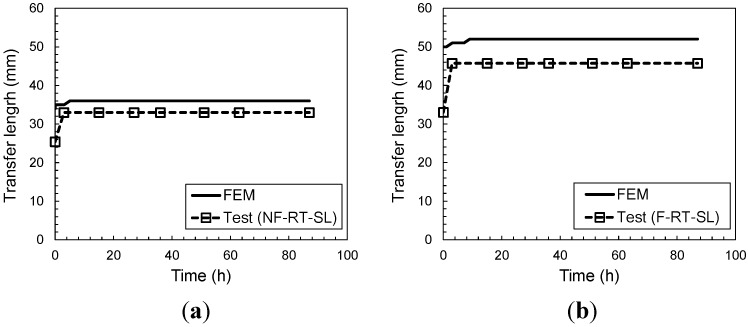
Transfer length during sustained loading at room temperature (23 °C) (**a**) with no manufactured flaw region; (**b**) with manufactured flaw region.

During the sustained loading period, transfer lengths were observed using photoelastic (P/E) coatings. Transfer length is normally defined as the length of the bonded CFRP sheet, which actively transfers stresses to the concrete substrate during loading. Therefore, transfer length was defined as the bonded length of CFRP over which non-zero strains were observed in the photoelastic strip. In terms of fringe, non-zero strain was indicated by the color black only. For the current purpose, photoelastic coatings used in these experiments can discern strain as small as 518 με. In this study, transfer length is defined as the length of CFRP over which longitudinal strains greater than 518 με were observed in the experiment. Figure 7 shows the numerical and experimental variation of transfer length over the 87 h of testing, for flawed and unflawed specimens with sustained load applied at room temperature. Most of the growth in transfer length occurred in the initial three hours of the experiment, whereas in the numerical simulation, the growth in transfer length occurred in the initial 10 h. It can be observed that the FE model captures the general trend of the time-dependent deformation. As can be seen, however, the numerically obtained transfer length does not exactly correlate with the experimental results. Results based on numerical analysis exceed those based on the experiments by 9%–13%. Even though this discrepancy could be attributed to a number of reasons, such as elastic modeling of the CFRP material or creep behavior of the epoxy used to attach the photoelastic coating, these results do indicate that the presence of a flaw affects the deformation of the CFRP (measured as transfer length).

**Figure 8:**
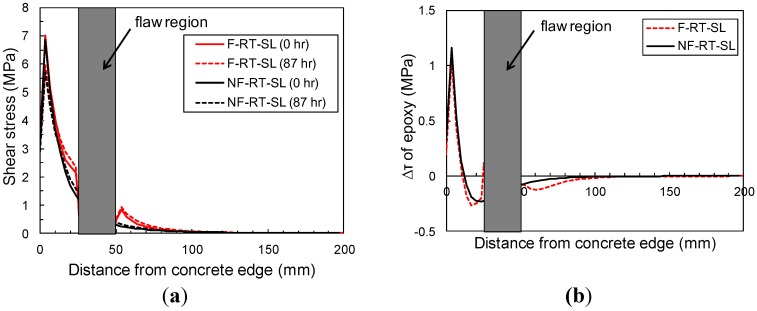
(**a**) Shear stress in epoxy layer during sustained loading period at room temperature; (**b**) Reduction of shear stress in epoxy layer between 0 and 87 h during sustained loading period.

Figure 8 shows the numerical time-dependent interface shear stress distribution at the midplane of the epoxy of single-lap specimens with and without a flaw at various times during sustained loading. The results have shown that the manufactured flaw can lead to an increase in the stress concentration when compared to the un-flawed specimen shown in Figure 8a. The shear stress concentrations are alleviated due to creep of the epoxy. In Figure 8b, a larger reduction of shear stress over time is seen in the flawed specimen than unflawed specimens, where the shear stress of the flawed specimen is greater than the unflawed specimen’s (at the edges of the manufactured flaw, 25 and 50 mm from the loaded end of the block). In interpreting these results, it should be kept in mind that, at this time, the model does not include time-dependent deformation or time-dependent damage in the crack band constitutive equations.

##### Pull-Off Loading Period

**Figure 9:**
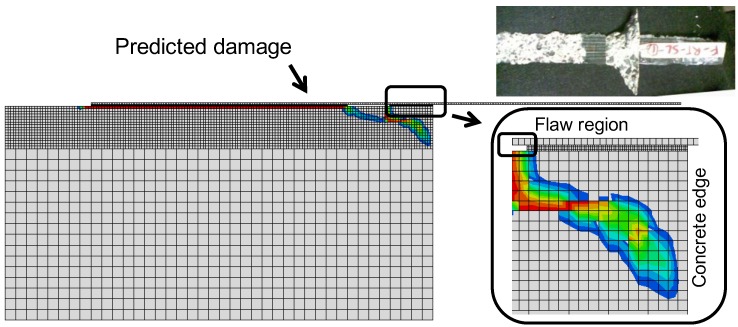
Experimental and numerical failure mode following sustained loading at room temperature. Photographs are experimental failure patterns (F-RT-SL).

Failure in the pull-off tests was sudden in both the experiments and the numerical simulations. In both cases, debonding began at the loaded end of the CFRP strip and progressed rapidly to the opposite end. Failure was at the epoxy/concrete interface in the experiments and the simulations, as shown in Figure 9. As can be seen, the failure mode obtained numerically correlates with the experimental results. Damage concentration occurs along the bond line for the region close to the loaded end of the FRP. In Figure 9, the specimen with a flaw shows damage distribution. As the free concrete edge is approached, the damage spreads to a large area, resulting in a concrete “tooth” attached to the CFRP strip at the loaded end.

**Figure 10:**
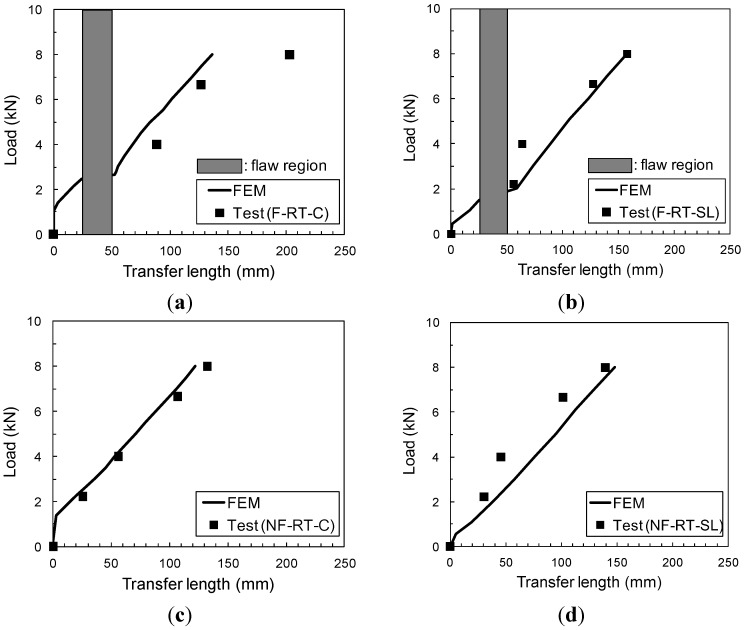
Experimental (Test) and numerically-simulated (FEM, Finite Element Method) transfer length *versus* applied load during pull-off testing. (**a**) F-RT-C; (**b**) F-RT-SL; (**c**) NF-RT-C; (**d**) NF-RT-SL.

Comparison of numerical and experimental transfer length *versus* applied load during pull-off testing, shown in Figure 10, indicates very good agreement for the specimens without flaws, with and without previous sustained loads at room temperature. Transfer length in specimens where there is no flaw tends to increase linearly with applied pull-off load in these load-controlled tests, which is also seen in the numerical results. In the specimens with flaws, the numerical results are in good agreement with the experimental results. Photoelastic coatings provided transfer length for all specimens, which is defined as the length of CFRP over which longitudinal strains greater than a 518 με threshold were observed in the experiment. Although permanent strain calculated numerically is observed in both specimens that have experienced sustained load at room temperature, the value of strain does not exceed the threshold until around 1.5 kN.

Experimental failure loads are listed in Table 5, along with results from numerical simulations. It is seen that failure loads calculated numerically are up to 13% higher than experimental loads. Despite these differences, the numerical results showed consistent trends in the individual effects of flaws and sustained loads.

From Table 5, it is evident that, numerically and experimentally, flawed specimens failed at slightly higher loads compared to unflawed specimens. This result can be attributed to the larger transfer length induced by the flaw. The cause of the increased failure loads is thought to be the increased participation of concrete in resisting the forces. Moreover, the presence of a flaw results in a concrete “tooth” failure as shown in Figure 9. It is predicted that specimens conditioned with sustained room temperature loading will suffer a slight reduction in pull-off strength. However, the experimental results were mixed.

**Table 5:** Predicted and measured failure loads for each specimen.

Specimen Identification	*P*_num_ (kN)	*P*_exp_ (kN)	*P*_num_/*P*_exp_	Effect of Flaw		Effect of Sustained Load	
				NUM	EXP	NUM	EXP
NF_RT_C (*a*)	9.66	8.54	1.13	–	–	–	–
F_RT_C (*b*)	9.79	8.80	1.11	1% (*b*/*a*) *	3% (*b*/*a*)	–	–
NF_RT_SL (*c*)	9.03	8.27	1.09	–	–	−6% (*c*/*a*)	−3% (*c*/*a*)
F_RT_SL (*d*)	9.93	9.16	1.08	10% (*d*/*c*)	10% (*d*/*c*)	1% (*d*/*b*)	4% (*d*/*b*)

* Notification example: *b*/*a* = 100(*b* − *a*)/*a*, where the letters are defined in column 1.

### 3.5. Sensitivity Study

In this section, numerical results from the developed FE models are presented to study the effects of various bonded-joint parameters such as epoxy thickness and test temperature on shear stress distribution along the midplane of the epoxy during sustained loading. Material properties presented in Table 1, epoxy creep data at HT, and the FE model geometry shown in Figure 4 are used as the baseline configuration without manufactured flaws. The parametric study was conducted varying one geometric or material parameter at a time.

#### 3.5.1. Sensitivity to Thickness of Epoxy

**Figure 11:**
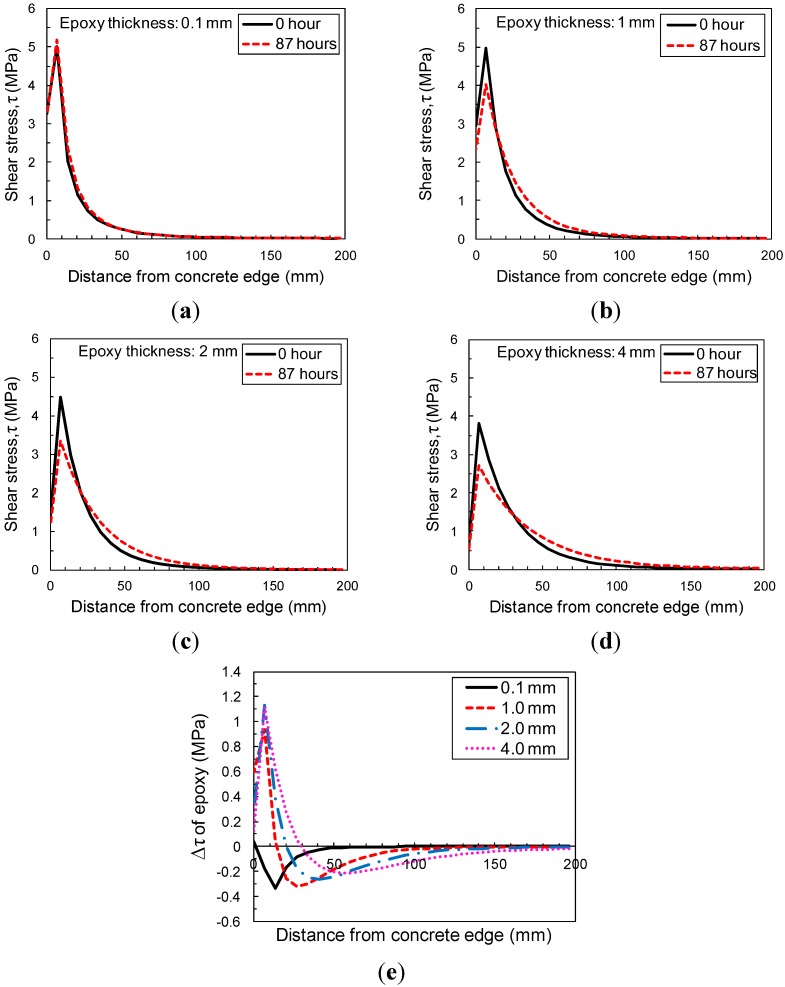
Sensitivity of epoxy thickness to shear stress distribution of epoxy at 40 °C: (**a**) with 0.1 mm thickness of epoxy; (**b**) with 1 mm thickness of epoxy; (**c**) with 2 mm thickness of epoxy; (**d**) with 4 mm thickness of epoxy; (**e**) reduction of shear stress in epoxy layer between 0 and 87 h during sustained loading period with different epoxy thicknesses.

Four epoxy thicknesses were chosen: 0.1 mm, 1.0 mm (baseline), 2 mm, and 4 mm. Figure 11 shows the effect of the epoxy thickness on shear stress at the midplane of the epoxy layer during an 87-h sustained loading period. The epoxy was modeled using the material properties (modulus and creep data) from the HT creep test (40 °C) described before. Numerical results suggest that the stress concentrations within epoxy interface relax with time. It is seen that increasing the thickness of the adhesive layer leads to a significant increase in amount of peak shear stress reduction over time due to creep and relaxation as shown in Figure 11d. Thus, it is apparent that creep can significantly affect bond stress distributions, particularly for thicker epoxy adhesive layers. This redistributing of stress in the transfer zone near the loaded end can cause a noticeable increase in displacement of the FRP relative to the concrete, which would be apparent as widening cracks in a concrete beam. On the other hand, the opposite behavior was observed in the case of 0.1 mm thickness of epoxy resin as shown in Figure 11a. A very thin layer (*i.e.*, 0.1 mm) of epoxy resin induces rare stress redistribution instead, with a slight increase in stress close to the loaded end. This opposite phenomenon can be attributed to the lack of epoxy along the interface, which can play a role in relaxing stress concentration near the loaded end because of its creep nature.

#### 3.5.2. Sensitivity to Temperature

**Figure 12:**
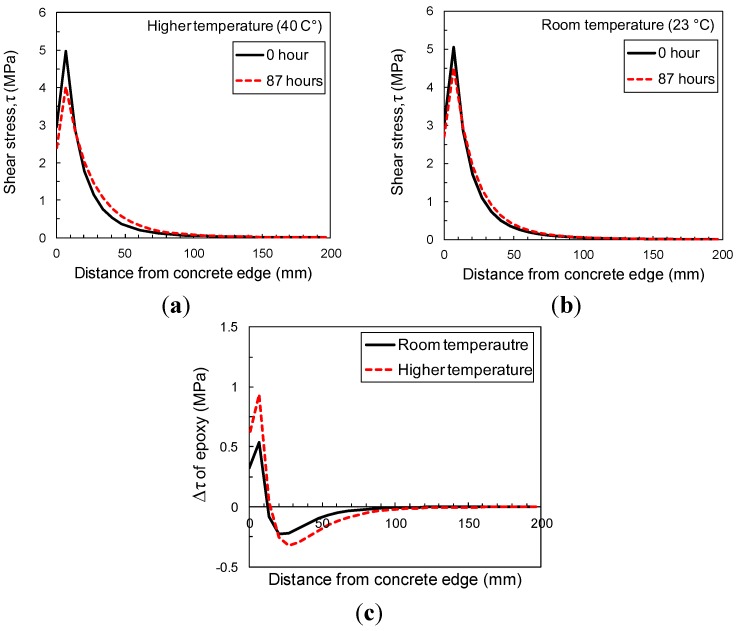
Sensitivity to temperature of shear stress distribution of 1-mm-thick epoxy (**a**) at higher temperature (40 °C); (**b**) at room temperature (23 °C); and (**c**) Reduction of shear stress in epoxy layer between 0 and 87 h during sustained loading period at different temperatures.

The effect of temperature during the sustained loading period is shown in Figure 12. Shear stress distributions are calculated immediately upon loading and after 87 h for modulus and creep properties at RT and HT. The maximum change in shear stress at HT is more than 70% higher than that at RT in Figure 12c, which could have important ramifications for the residual pull-off strength of the FRP/concrete joint after sustained loading in elevated temperatures. More research is recommended on the evolving strength of the interfacial region during sustained loads at elevated temperatures.

## 4. Conclusions

The behavior of concrete blocks with bonded CFRP strips under the effects of sustained load, elevated temperature, and flaws were simulated using finite element analysis. The following conclusions can be derived from this study:

1. Based on the experimental data, the proposed model has the ability to estimate the effects of flaws and sustained loading in terms of the pull-off strength.
2. The presence of a manufactured flaw in the specimen under sustained loading extends the transfer length, which also increased the pull-off strength. It appears that existing flaws in the specimens in this study engage a larger transfer length, which also demands greater participation from the concrete substrate.
3. Following the sustained loading period, the pull-off strength of the single-lap shear specimen decreased, when compared to the specimens not subjected to sustained loading.
4. The numerical model used in this study predicts, with good correlation with experimental results, the failure mode and pull-off behavior of the specimen after experiencing sustained loading.
5. The results of sensitivity studies confirm that the specimen’s creep response is sensitive to geometric and temperature-dependent material parameters. While the parameters of the proposed model, which are obtained based on creep tests, can be acceptable to estimate the pull-off behavior of the specimens experiencing sustained loading, more experimental studies are needed to confirm the validity of these parameters in single-lap specimens during sustained loading at different temperatures and in pull-off tests to failure following such conditioning.
